# The advanced lung cancer inflammation index predicts short and long-term outcomes in patients with colorectal cancer following surgical resection: a retrospective study

**DOI:** 10.7717/peerj.10100

**Published:** 2020-10-08

**Authors:** Hailun Xie, Shizhen Huang, Guanghui Yuan, Jiaan Kuang, Ling Yan, Lishuang Wei, Shuangyi Tang, Jialiang Gan

**Affiliations:** 1 Department of Colorectal and Anal Surgery, The First Affiliated Hospital, Guangxi Medical University, Nanning, Guangxi, P.R. China; 2 Department of Respiratory Medicine, The First Affiliated Hospital, Guangxi Medical University, Nanning, Guangxi, P.R. China; 3 Department of Pharmacy, The First Affiliated Hospital, Guangxi Medical University, Nanning, Guangxi, P.R. China

**Keywords:** Colorectal cancer, Advanced lung cancer inflammation index, Complication, Prognosis

## Abstract

**Background and Purpose:**

Several studies have proposed that the advanced lung cancer inflammation index (ALI), a new inflammation-related index, can be used for the prognosis assessment of various malignancies. However, few studies have reported its prognostic value in colorectal cancer (CRC). Therefore, this study explored the relationship between ALI and outcomes in CRC patients.

**Methods:**

A total of 662 CRC patients who underwent surgery between 2012 and 2014 were included. The ALI was defined as: body mass index × serum albumin/neutrophil to lymphocyte ratio. The X-tile program identified the optimal cut-off value of ALI. Logistic regression analyses determined factors affecting postoperative complications. The Kaplan–Meier method and Cox proportional hazards analyses evaluated potential prognostic factors.

**Results:**

The optimal cut-off of ALI in males and females were 31.6 and 24.4, respectively. Low-ALI was an independent risk factor for postoperative complications in CRC patients (odds ratio: 1.933, 95% CI [1.283–2.911], *p* = 0.002). Low-ALI groups also had significantly lower progression-free survival (PFS) and overall survival (OS), when compared with the high-ALI group, especially at advance tumor stages. Using multivariate analysis, ALI was determined as an independent prognostic factor for PFS (hazard ratio: 1.372, 95% CI [1.060–1.777], *p* = 0.016) and OS (hazard ratio: 1.453, 95% confidence interval: 1.113–1.898, *p* = 0.006).

**Conclusion:**

ALI is an independent predictor of short and long-term outcomes in CRC patients, especially at advance tumor stages. The ALI-based nomograms can provide accurate and individualized prediction of postoperative complication risk and survival for CRC patients.

## Introduction

Colorectal cancer (CRC) is the third most common malignant tumor and the second leading cause of cancer-related death worldwide. According to Global Cancer Epidemiological Statistics, approximately 1.8 million people are diagnosed with CRC, and 881,000 patients die annually from the disease (Bray et al., 2018). In China, CRC is one of the five most common tumors, with a high morbidity and mortality rate (Chen et al., 2016). With the advancement of therapeutic treatments such as surgery, chemotherapy, immunotherapy and radiotherapy, 5- and 10-year survival rates can reach 58%–65%, but the survival rate of patients with tumor recurrence and metastasis can be reduced to 5%–10%(Miller et al., 2016). Therefore, determining effective prognostic indicators for patient survival can help clinicians adopt better prevention and treatment methods, thereby reducing cancer-related mortality.

There is increasing research that tumor-associated inflammation plays crucial roles in the development and progression of tumor (Mantovani et al., 2008). During tumorigenesis and development, pro-inflammatory cytokines and inflammatory cells are activated, which promotes the formation of new lymphatic and blood vessels, thus developing a tumor microenvironment conducive to the growth and differentiation of tumor cells. Tumorigenesis also destroy immune cell function, which makes tumor cells more prone to invasion and metastasis (Balkwill & Mantovani, 2001; Coussens & Werb, 2002; Mantovani et al., 2008). Consequently, inflammation-related factors promise to be valuable prognostic biomarkers for tumor. In addition, Malnutrition is associated with both complications and long-term outcomes (Alifano et al., 2014; Schwegler et al., 2010). CRC patients are more likely to suffer from malnutrition due to comorbidities such as local obstruction, bleeding, and perforation. Malnutrition increases the risk of surgery and prolongs hospital stays, and postoperative mortality of patients is greatly increased.

Recently, several studies have found that the Advanced Lung Cancer Inflammation Index (ALI), a new inflammation-related index, can be used for the prognosis assessment of various malignancies, including metastatic non-small cell lung cancer (Jafri, Shi & Mills, 2013), small cell lung cancer (Kim et al., 2016), and diffuse large B-cell lymphoma (Park et al., 2017). ALI was commonly used to assess the prognosis of patients with lung cancer. Recently, it has been gradually found that ALI is also applicable to gastrointestinal cancer. Feng, Huang & Chen (2014). reported in 2014 that ALI is a useful prognostic factor for patients with esophageal squamous cell carcinoma. Shibutani et al. (2019). based on 159 unresectable metastatic CRC patients reported that ALI is an effective prognostic marker in patients with unresectable metastatic CRC as well as in patients with lung cancer. Gastrointestinal cancer was more prone to malnutrition due to cancer consumption, obstruction, and bleeding. In addition, inflammation is considered to be an important factor in the occurrence and development of gastrointestinal cancer. ALI assembled multiple inflammatory and nutritional indicators can be considered as a potential prognostic factor for gastrointestinal cancer. However, no large-scale study has yet investigated the prognostic value of ALI in CRC patients following surgical resection; therefore, we explored the relationship between ALI and the short- and long-term outcomes of CRC patients following surgical resection using a single-center retrospective analysis.

## Materials & Methods

### Population and laboratory data

A total of 622 CRC patients who underwent operation in the Department of Colorectal and Anal Surgery, the First Affiliated Hospital of Guangxi Medical University from 2012 to 2014 were included. This study has excluded patients with preoperative neoadjuvant radiotherapy and chemotherapy. All the included patients had received surgical resection of the primary tumor. Basic information on patients included gender, age, height, weight. Preoperative laboratory serological measurements included neutrophil and lymphocyte counts, albumin (hypoproteinemia as defined by albumin <  35 g/L) and serum CEA levels (normal <  5.00 ng/ml; high, ≧5.00 ng/ml). All preoperative laboratory serological measurements were taken within one week prior to surgery. Tumor characteristics included pathological tumor-node-metastasis stage (pTNM stage), pathological tumor stage (pT stage), pathological node stage (pN stage), clinical distant metastasis, tumor location, tumor size, perineural invasion, vascular invasion, pathological type, and histological grade. Other information included the surgical approach, postoperative complications and Clavien–Dindo classification. The NLR (neutrophil/lymphocyte ratio) was calculated as the absolute neutrophil count divided by the absolute lymphocyte count (The median NLR was 2.23, which was divided into high and low NLR groups). The body mass index (BMI) was calculated as the body weight (kg) divided by the height squared (m^2^) (low <18.5; normal 18.5–24 and high ≧24). ALI was defined as BMI (kg/m^2^) × albumin (g/dL)/NLR. This study was approved by the Hospital Ethics Committee of the First Affiliated Hospital of Guangxi Medical University, Guangxi, China. The approval number: 2019(KY-Z-022). All patients in our study have written informed consent.

### Postoperative follow-up

Postoperative follow-up was implemented every three months for the first two years, and then every six months thereafter. The last follow-up was September 1, 2019. Follow-up procedures included serological examination, abdominal plain film, upper abdominal computed tomography (CT) and colonoscopy. The overall survival (OS) is the time interval from the date of surgery to death, or the last follow-up. The progression-free survival (PFS) is defined as the time interval between surgery and disease recurrence, death or the last follow-up of a patient who did not relapse.

### Statistical analysis

With 5-year overall survival status and 5-year overall survival time as events, X-tile program was used to determine the optimal cut-off value of ALI. The chi-square test or Fisher’s exact test was used to compare the clinicopathological variables. Logistic regression analysis was performed to determine the factors affecting postoperative total complications. Kaplan–Meier method was used to estimate survival rates and log-rank test was used for comparison. Cox proportional risk regression analysis were used to validate prognostic factors associated with PFS and OS. The areas under the curve (AUCs) are used to compare the prognostic ability of ALI and its components based on their 5-year PFS and 5-year OS. The ALI-based nomograms were constructed based on the results of multivariate analysis. The discrimination ability of the nomograms was evaluated by Consistency-index (C-index) and calibration curve. The IBM SPSS version 24.0 and R version 3.5.3 was used to analyze the data, a *p* value of <0.05 was considered statistically significant.

## Results

### Baseline characteristics

According to the X-tile program (Camp, Dolled-Filhart & Rimm, 2004), the optimal cut-off value for ALI for male patients was 31.6, and for female was 24.4. Using these cut-off values, 239 (36.1%) patients had low ALI scores, and 423 (63.9%) patients had high ALI scores. The baseline characteristics of the 662 CRC patients were shown (Table 1). 69 (10.4%) patients were deemed to have high-grade histological grade, including 11 cases of signet ring cell carcinoma and 58 cases of mucinous adenocarcinoma. Patients with TNM stage I numbered 125 (22.8%), the TNM stage II group had 215 (32.5%) patients, the TNM stage III group had 247 (37.3%) patients, and the TNM stage IV group had 75 (7.4%) patients.

**Table 1:** The relationships between the ALI (and its components) and clinicopathological factors of CRC patients.

Features	Case No. (%)	ALI			BMI (kg/m^2^				Albumin(g/dl)			NLR		
		Low (%)	High (%)	*p*	Low (%)	Normal (%)	High (%)	*p*	Low (%)	Normal (%)	*p*	Low (%)	High (%)	*p*
Gender				<0.001				0.168			0.224			0.055
Male	408(61.6)	183(76.6)	225(53.2)		61(59.2)	230(59.6)	117(67.6)		129(65.2)	279(60.1)		192(58.0)	216(65.3)	
Female	254(38.4)	56(23.4)	198(46.8)		42(40.8)	156(40.4)	56(32.4)		69(34.8)	185(39.9)		139(42.0)	115(34.7)	
Age (Years)				0.008				0.130			<0.001			0.276
<60	342(51.7)	107(44.8)	235(55.6)		50(48.5)	212(54.9)	80(46.2)		77(38.9)	265(57.1)		178(53.8)	164(49.5)	
≥60	320(48.3)	132(55.2)	188(44.4)		53(51.5)	174(45.1)	93(53.8)		121(61.1)	199(42.9)		153(46.2)	167(50.5)	
pT stage				0.324				0.789			0.440			0.788
T1-2	167(25.2)	55(23.0)	112(26.5)		25(24.3)	95(24.6)	47(27.2)		46(23.2)	121(26.1)		85(25.7)	82(24.8)	
T3-4	495(74.8)	184(77.0)	311(73.5)		78(75.7)	291(75.4)	126(72.8)		152(76.8)	343(73.9)		246(74.3)	249(75.2)	
pN stage				0.363				0.782			0.598			0.357
N0	368(55.6)	130(54.4)	238(56.3)		61(59.2)	217(56.2)	90(52.0)		116(58.6)	252(54.3)		181(54.7)	187(56.5)	
N1	187(28.2)	64(26.8)	123(29.1)		27(26.2)	109(28.2)	51(29.5)		52(26.3)	135(29.1)		101(30.5)	86(26.0)	
N2	107(16.2)	45(18.8)	62(14.7)		15(14.6)	60(15.5)	32(18.5)		30(15.2)	77(16.6)		49(14.8)	58(17.5)	
Clinical distant metastasis				<0.001				0.161			0.022			<0.001
No	587(88.7)	191(79.9)	396(93.6)		87(84.5)	341(88.3)	159(91.9)		167(84.3)	420(90.5)		310(93.7)	277(83.7)	
Yes	75(11.3)	48(20.1)	27(6.4)		16(15.5)	45(11.7)	14(8.1)		31(15.7)	44(9.5)		21(6.3)	54(16.3)	
Tumor location				<0.001				0.506			<0.001			<0.001
Rectal	346(52.3)	103(43.1)	243(57.4)		49(47.6)	208(53.9)	89(51.4)		78(39.4)	268(57.8)		196(59.2)	150(45.3)	
Colon	316(47.7)	136(56.9)	180(42.6)		54(52.4)	178(46.1)	84(48.6)		120(60.6)	196(42.2)		135(40.8)	181(54.7)	
Tumor size				<0.001				0.121			<0.001			0.001
<5 cm	329(49.7)	92(38.5)	237(56.0)		42(40.8)	195(50.5)	92(53.2)		69(34.8)	260(56.0)		186(56.2)	143(43.2)	
≥5 cm	333(50.3)	147(61.5)	186(44.0)		61(59.2)	191(49.5)	81(46.8)		129(65.2)	204(44.0)		145(43.8)	188(56.8)	
Perineural invasion				0.640				0.249			0.544			0.787
Negative	602(90.9)	219(91.6)	383(90.5)		94(91.3)	356(92.2)	152(87.9)		178(89.9)	424(91.4)		302(91.2)	300(90.6)	
Positive	60(9.1)	20(8.4)	40(9.5)		9(8.7)	30(7.8)	21(12.1)		20(10.1)	40(8.6)		29(8.8)	31(9.4)	
Vascular invasion				0.349				0.271			0.558			0.282
Negative	560(84.6)	198(82.8)	362(85.6)		92(89.3)	326(84.5)	142(82.1)		165(83.3)	395(85.1)		285(86.1)	275(83.1)	
Positive	102(15.4)	41(17.2)	61(14.4)		11(10.7)	60(15.5)	31(17.9)		33(16.7)	69(14.9)		46(13.9)	56(16.9)	
Pathological type				0.268				0.365			0.069			0.037
Protrude type	131(19.8)	55(23.0)	76(18.0)		25(24.3)	77(19.9)	29(16.8)		50(25.3)	81(17.5)		53(16.0)	78(23.6)	
Infiltrating type	77(11.6)	25(10.5)	52(12.3)		15(14.6)	40(10.4)	22(12.7)		22(11.1)	55(11.9)		37(11.2)	40(12.1)	
Ulcerative type	454(68.6)	159(66.5)	295(69.7)		63(61.2)	269(69.7)	122(70.5)		126(63.6)	328(70.7)		241(72.8)	213(64.4)	
Histological grade				0.809				0.342			0.629			0.899
High-grade	69(10.4)	24(10.0)	45(10.6)		7(6.8)	45(11.7)	17(6.9)		19(9.5)	50(10.8)		34(10.3)	35(10.6)	
Low-grade	593(89.6)	215(90.0)	378(89.4)		96(93.2)	341(88.3)	156(90.2)		180(90.5)	413(89.2)		297(89.7)	296(89.4)	
CEA				<0.001				0.832			0.004			<0.001
<5 ng/ml	390(58.9)	107(44.8)	283(66.9)		63(61.2)	224(58.0)	103(59.5)		100(50.5)	290(62.5)		226(68.3)	164(49.5)	
≥5 ng/ml	272(41.1)	132(55.2)	140(33.1)		40(38.8)	162(42.0)	70(40.5)		98(49.5)	174(37.5)		105(31.7)	167(50.5)	

### Association of ALI and its components with clinicopathological factors

The relationship between preoperative ALI and other clinicopathological factors is shown in Table 1. Male, advanced age, clinical distant metastasis, colon cancer, large tumor, high CEA levels were significantly associated with the low ALI group. There were no significant differences between the two ALI groups with tumor-related factors such as pT stage, pN stage, perineural invasion, vascular invasion, histological grade, and pathological type.

### Associations of ALI with postoperative complications

The classification of complications was shown in Table 2. 127 patients (19.2%) suffered from clinically relevant complications after surgery, including postoperative bowel obstruction (12 cases), anastomotic leak (11 cases), gastrointestinal problems (17 cases), wound problems (55 cases), pulmonary complications (16 cases), and other complications (16 cases). Patients with low ALI scores had a higher incidence of total complications, grade I and grade II complications, when compared with the high ALI group. In univariate logistic analyses, advanced age, low-ALI, open surgery and high CEA levels were correlated with postoperative total complications, but in multivariate logistic analyses, only age (odds ratio: 1.627, 95% CI [1.090–2.429], *p* = 0.017) and ALI (odds ratio: 1.933, 95% CI [1.283–2.911], *p* = 0.002) were independent factors affecting postoperative total complications (Table 3).

**Table 2:** Details of postoperative complications according to clavien-dindo classification.

Grade	Total (*n* = 662)	Low-ALI (*n* = 239)	High-ALI (*n* = 423)	X^2^	*p*
Total complications	127 (19.2%)	66 (27.6%)	61 (14.4%)	17.148	<0.001
Grade I	56 (8.5%)	29 (12.1%)	27 (6.4%)	6.522	0.011
Grade II	54 (8.2%)	28 (11.7%)	26 (6.1%)	6.322	0.012
Grade III	11 (1.7%)	5 (2.1%)	6 (1.4%)	0.424	0.515
Grade IV	5 (0.7%)	3 (1.3%)	2 (0.5%)	1.247	0.264
Grade V	1 (0.1%)	1 (0.4%)	0 (0.0%)	1.773	0.183

**Table 3:** Univariate and multivariate Logistic regression analysis of complications in CRC patients.

Feature	Univariate analysis		Multivariate analysis	
	OR (95% CI)	*p*	OR (95% CI)	*p*
Gender (Female)	0.931 (0.624–1.389)	0.726		
Age (≥60)	1.777 (1.199–2.634)	0.004	1.627 (1.090–2.429)	0.017
ALI (Low)	2.264 (1.529–3.352)	<0.001	1.933 (1.283–2.911)	0.002
pT stage (T3-4)	1.175 (0.744–1.856)	0.490		
pN stage		0.770		
N0	1.000			
N1	1.142 (0.736–1.772)			
N2	0.936 (0.859–1.639)			
Clinical distant metastasis (Yes)	1.505 (0.859–2.636)	0.153		
Tumor location (Colon)	0.902 (0.612–1.330)	0.604		
Tumor size (≧5 cm)	1.044 (0.709–1.538)	0.826		
Perineural invasion (Positive)	1.458 (0.785–2.709)	0.232		
Vascular invasion (Positive)	1.365 (0.824–2.262)	0.227		
Pathological type		0.167		
Protrude type	1.000			
Infiltrating type	1.088 (0.497–2.385)			
Ulcerative type	1.581 (0.925–2.702)			
Histological grade (High-grade)	1.435 (0.799–2.578)	0.226		
Surgical approach (Open)	1.603 (1.078–2.384)	0.020	1.373 (0.911–2.068)	0.130
CEA (High)	1.597 (1.083–2.356)	0.018	1.313 (0.911–2.068)	0.186

### Associations of ALI with survival outcomes

199 (30.1%) patients suffered recurrence and metastasis, and 248 (37.5%) patients died (179 patients died from recurrence and metastasis, and 69 died from other causes). The median follow-up time was 63 months (1–80 months). Univariate analyses showed that patients with low ALI, T3–4 stage, pN stage, clinical distant metastasis, large tumor size, positive perineural invasion, positive vascular invasion, high-grade histological grade, open surgery and high serum CEA levels, had lower PFS. The same result was also found for OS (Table 4). Multivariable analyses showed that only ALI, pN stage, clinical distant metastasis, histological grade and preoperative CEA levels were independent prognostic factors for PFS and OS.

**Table 4:** Univariate and multivariate survival analysis of clinicopathological characteristics in CRC patients.

Feature	Progression-free survival				Overall survival			
	Univariate		Multivariate		Univariate		Multivariate	
	HR (95% CI)	*p*	HR (95% CI)	*p*	HR (95% CI)	*p*	HR (95% CI)	*p*
Gender (Female)	0.906 (0.706–1.161)	0.435			0.874 (0.674–1.134)	0.311		
Age (≥60)	1.144 (0.900–1.453)	0.272			1.257 (0.980–1.614)	0.072		
ALI (Low)	1.779 (1.399–2.262)	<0.001	1.372 (1.060–1.777)	0.016	1.843 (1.436–2.366)	<0.001	1.453 (1.113–1.898)	0.006
pT stage (T3-4)	2.339 (1.671–3.274)	<0.001	1.387 (0.968–1.988)	0.075	2.512 (1.749–3.607)	<0.001	1.461 (0.992–2.150)	0.055
pN stage		<0.001		<0.001		<0.001		<0.001
N0	1.000		1.000		1.000		1.000	
N1	1.679 (1.255–2.244)		1.374 (1.016–1.859)		1.656 (1.222–2.246)		1.382 (1.007–1.896)	
N2	4.508 (3.371–6.029)		2.939 (2.133–4.050)		4.555 (3.374–6.149)		3.001 (2.158–4.173)	
Clinical distant metastasis (Yes)	5.794 (4.372–7.679)	<0.001	3.414 (2.481–4.697)	<0.001	5.386 (4.027–7.202)	<0.001	3.231 (2.331–4.479)	<0.001
Tumor location (Colon)	1.028 (0.808–1.306)	0.824			0.999 (0.779–1.282)	0.996		
Tumor size (≥5 cm)	1.307 (1.028–1.663)	0.029	0.997 (0.774–1.284)	0.981	1.347 (1.049–1.731)	0.020	1.029 (0.791–1.339)	0.831
Perineural invasion (Positive)	1.588 (1.103–2.286)	0.013	1.141 (0.754–1.729)	0.532	1.482 (1.006–2.184)	0.047	1.086 (0.702–1.681)	0.711
Vascular invasion (Positive)	1.875 (1.407–2.500)	<0.001	1.082 (0.773–1.513)	0.647	1.794 (1.329–2.421)	<0.001	1.042 (0.737–1.473)	0.815
Macroscopic type		0.432				0.333		
Protrude type	1.000				1.000			
Infiltrating type	1.214 (0.773–1.906)				1.322 (0.829–2.108)			
Ulcerative type	1.236 (0.896–1.706)				1.280 (0.911–1.799)			
Histological grade (High-grade)	1.667 (1.186–2.341)	0.003	1.495 (1.052–2.125)	0.025	1.802 (1.275–2.547)	0.001	1.622 (1.132–2.323)	0.008
Surgical approach (Open)	1.340 (1.051–1.709)	0.018	1.022 (0.788–1.326)	0.868	1.340 (1.041–1.725)	0.023	1.010 (0.771–1.324)	0.942
CEA (High)	2.060 (1.620–2.621)	<0.001	1.421 (1.100–1.837)	0.007	2.093 (1.629–2.689)	<0.001	1.481 (1.138–1.928)	0.004
Postoperative chemotherapy (Yes)	1.065 (0.838–1.353)	0.609			1.071 (0.835–1.375)	0.588		

**Figure 1:**
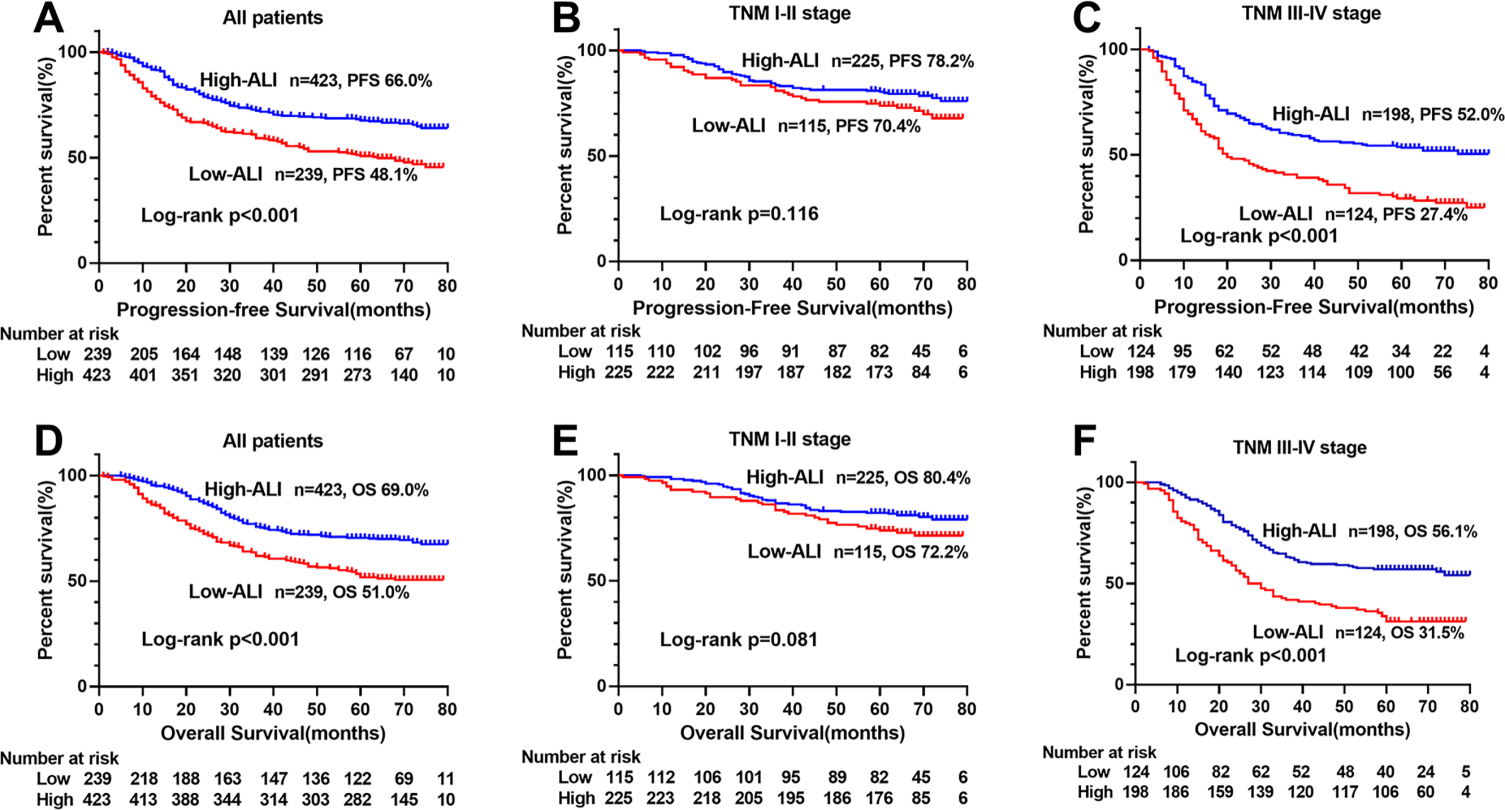
Kaplan–Meier survival curves of low-ALI and high-ALI groups of CRC patients based on TNM stages. (A) Kaplan–Meier progression-free survival curves of all patients; (B) Kaplan–Meier progression-free survival curves of TNM I-II stage patients; (C) Kaplan–Meier progression-free survival curves of TNM III-IV stage patients; (D) Kaplan–Meier overall survival curves of all patients; (E) Kaplan–Meier overall survival curves of TNM I-II stage patients; (F) Kaplan–Meier overall survival curves of TNM III-IV stage patients. Abbreviations: ALI, Advanced Lung Cancer Inflammation Index; PFS, progression-free survival; OS, overall survival.

**Figure 2:**
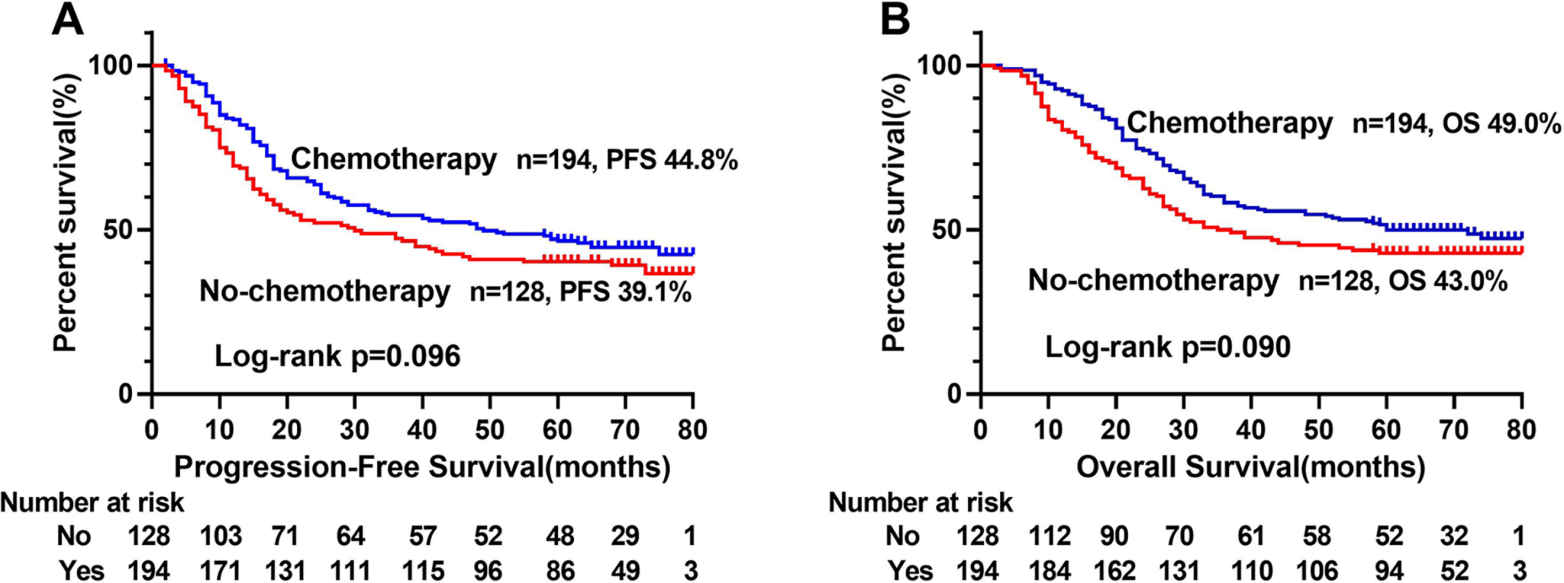
Kaplan–Meier survival curves of postoperative chemotherapy of CRC patients with TNM III-IV stage. (A) Kaplan–Meier progression-free survival curves; (B), Kaplan–Meier overall survival curves. Abbreviations: PFS, progression-free survival; OS, overall survival.

**Figure 3:**
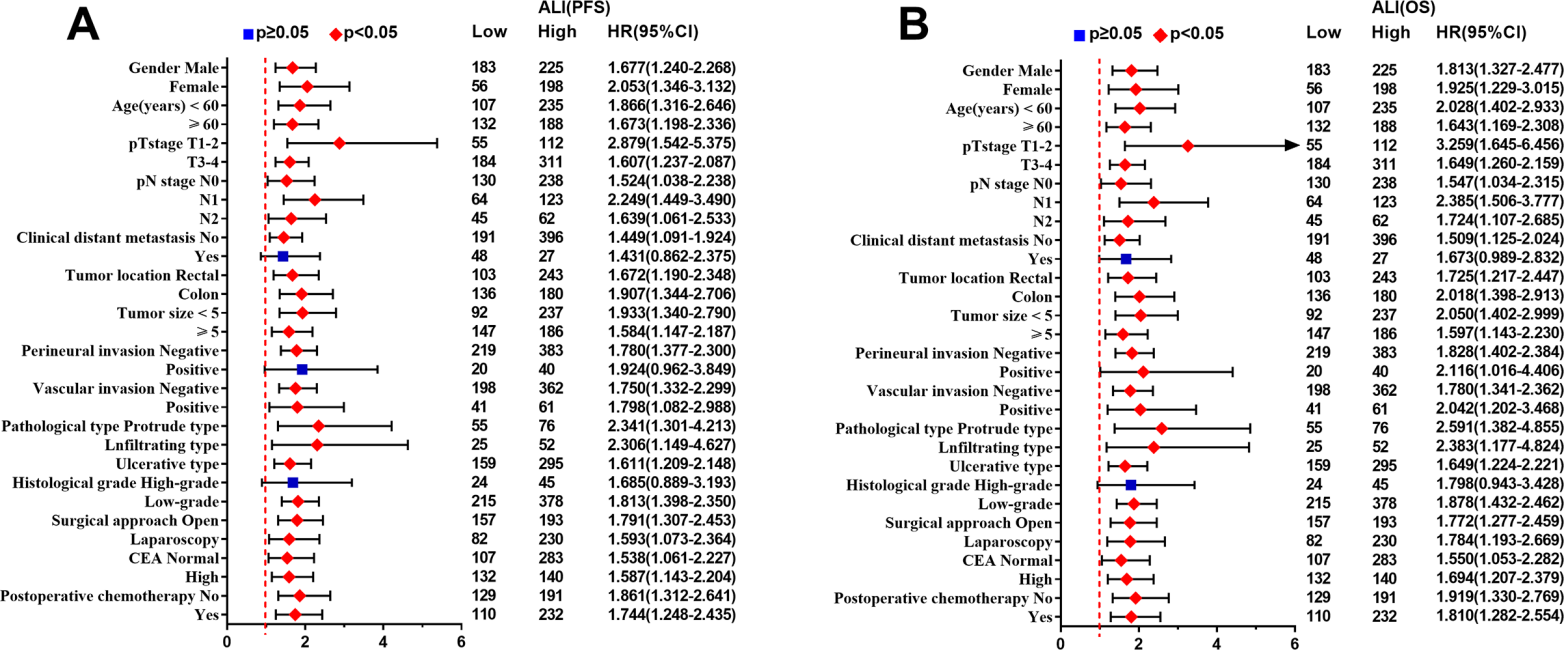
Subgroup survival analysis of ALI in CRC patients. (A) Subgroup progression-free survival analysis; (B) Subgroup overall survival analysis. Abbreviations: ALI, Advanced Lung Cancer Inflammation Index; PFS, progression-free survival; OS, overall survival.

The Kaplan–Meier survival curve showed that patients with low ALI scores were significantly lower than those with high ALI scores in terms of PFS (48.1% vs. 66.0%, *p* < 0.001) and OS (51.0% vs. 69.0%, *p* < 0.001) (Figs. 1A, 1D). Further stratified analyses based on TNM stages, showed significant differences in PFS and OS between high- and low-ALI groups in patients with TNM III–IV stage (Figs. 1C, 1F), while there were no significant differences in patients with TNM I–II stage (Figs. 1B, 1E). In addition, postoperative chemotherapy could improve survival rates of CRC patients with TNM III-IV stage to some extent, but there was no statistical significance (Figs. 2A, 2B). In the subgroup analyses, we found that low ALI scores in most subgroups was an independent risk factor affecting the PFS (Fig. 3A) and OS (Fig. 3B) of CRC patients.

### Predictive ability of prognosticators

**Figure 4:**
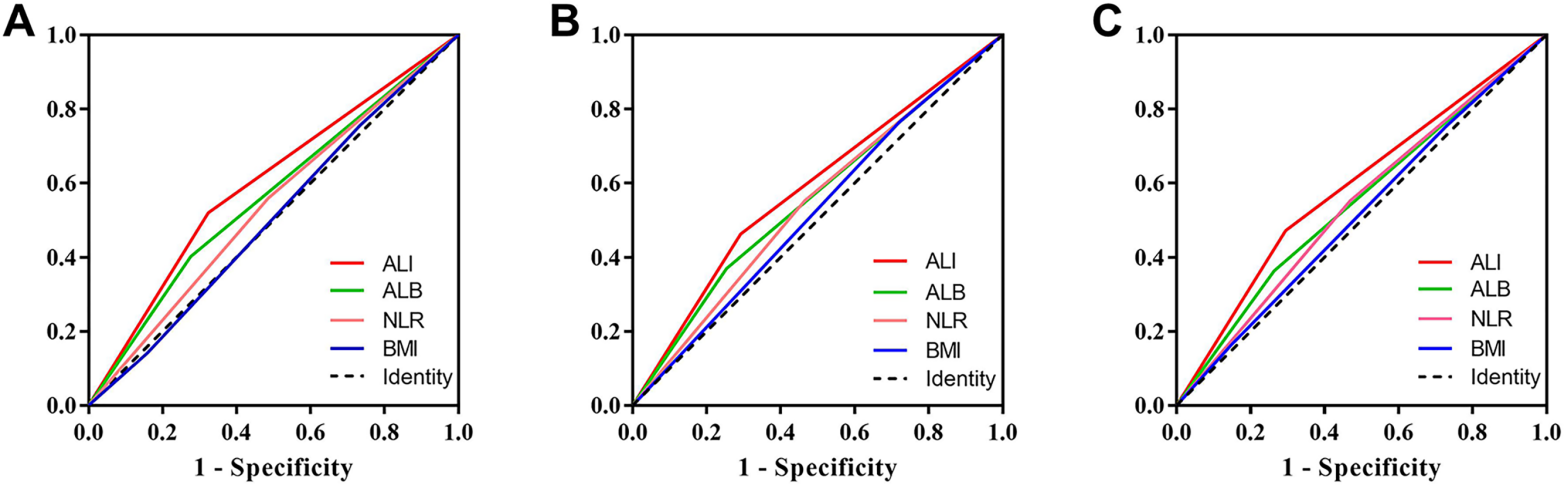
Area under the receiver operating characteristic curves of ALI and its components for the prediction of prognosis. (A) Complications; (B) progression-free survival; (C) overall survival. Abbreviations: ALI, Advanced Lung Cancer Inflammation Index; ALB, albumin; NLR, neutrophils/lymphocytes ratio; BMI, Body Mass Index.

**Figure 5:**
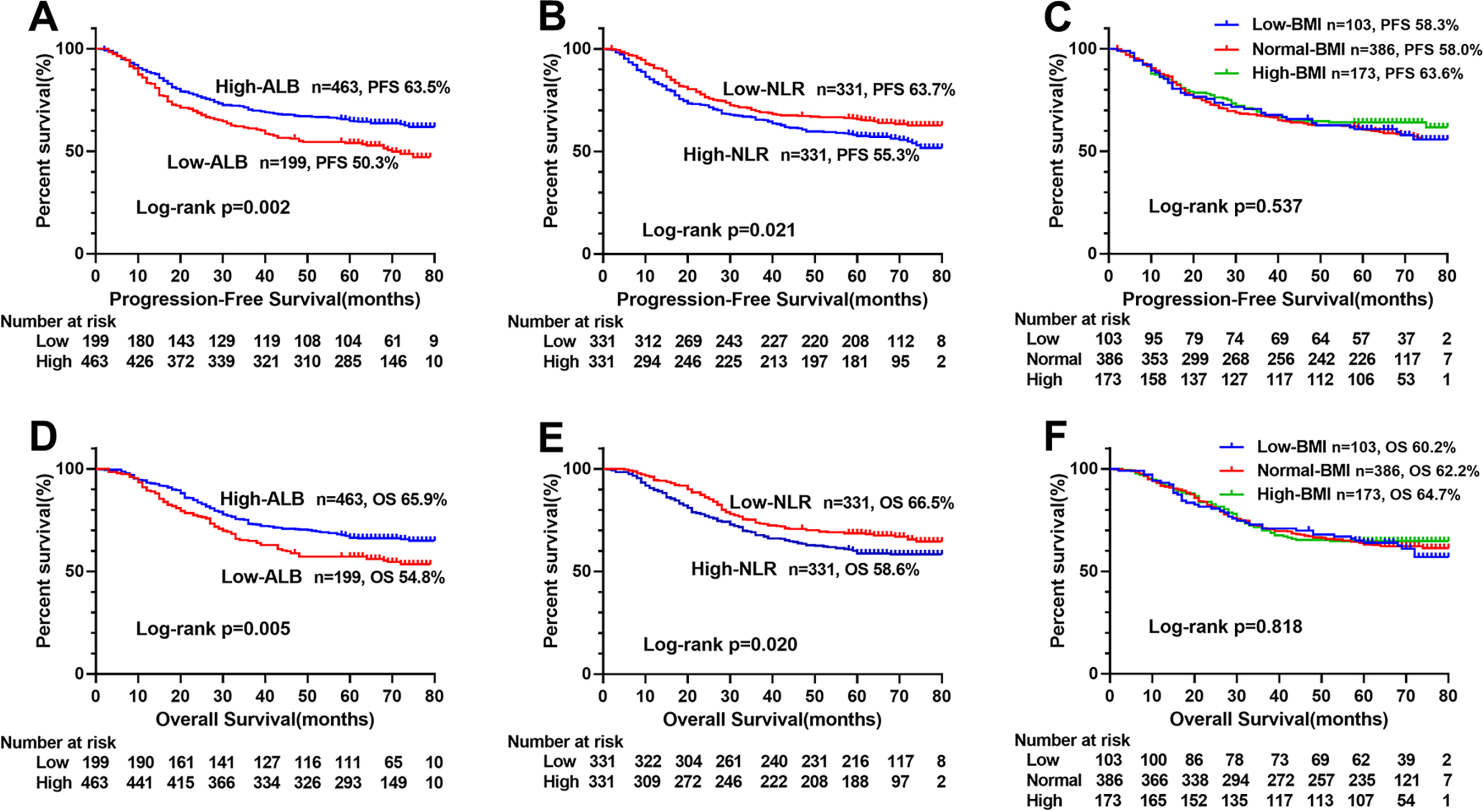
Kaplan–Meier survival curves of each components of ALI in CRC patients. (A) Kaplan–Meier progression-free survival curves of ALB; (B), Kaplan–Meier progression-free survival curves of NLR; (C), Kaplan–Meier progression-free survival curves of BMI; (D), Kaplan–Meier overall survival curves of ALB; E, Kaplan–Meier overall survival curves of NLR; (F), Kaplan–Meier overall survival curves of BMI. Abbreviations: ALI, Advanced Lung Cancer Inflammation Index; ALB, albumin; NLR, neutrophil/lymphocyte ratio; BMI, body mass index; PFS, progression-free survival; OS, overall survival.

We compared the prognostic ability of ALI and its components using the AUCs based on their complications (Fig. 4A), 5-year PFS (Fig. 4B) and 5-year OS (Fig. 4C). The result indicated the prognostic ability of ALI (complications, 0.598, 95% CI [0.542–0.654], *p* < 0.001; PFS, 0.585, 95% CI [0.541–0.630], *p* < 0.001; OS, 0.589, 95% CI [0.543–0.634], *p* < 0.001) was better than its components as follows: albumin (complications, 0.563, 95% CI [0.506–0.619], *p* = 0.029; PFS, 0.558, 95% CI [0.513–0.603], *p* = 0.012; OS, 0.550, 95% CI [0.504–0.596], *p* = 0.032), NLR (complications, 0.537, 95% CI [0.481–0.592], *p* = 0.200; PFS, 0.544, 95% CI [0.499–0.589], *p* = 0.055; OS, 0.542, 95% CI [0.497–0.587], *p* = 0.071), and BMI (complications, 0.503, 95% CI [0.448–0.558], *p* = 0.925; PFS, 0.522, 95% CI [0.477–0.566], *p* = 0.344; OS, 0.516, 95% CI [0.471–0.562], *p* = 0.487). We also compared the Kaplan–Meier survival curve of ALI and its components, we found that ALI (Figs. 1A, 1D), ALB (Figs. 5A, 5D), and NLR (Figs. 5B, 5E) can distinguish patients with poor prognosis, while BMI (Figs. 5C, 5F) cannot. Compared with ALB and NLR, ALI could separate the survival curve more effectively.

### Construction of ALI-based nomograms

**Figure 6:**
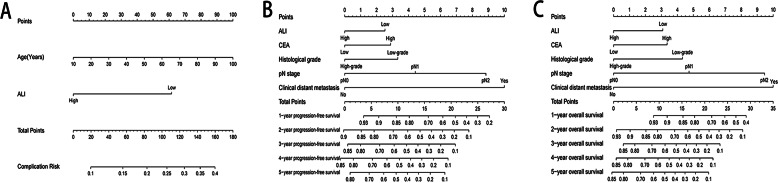
Construction of ALI-based nomograms in CRC patients. (A) ALI-based nomograms of complication risk; (B) ALI-based nomograms of progression-free survival; (C) ALI-based nomograms of overall survival. Abbreviations: ALI, Advanced Lung Cancer Inflammation Index; PFS, progression-free survival; OS, overall survival.

**Figure 7:**
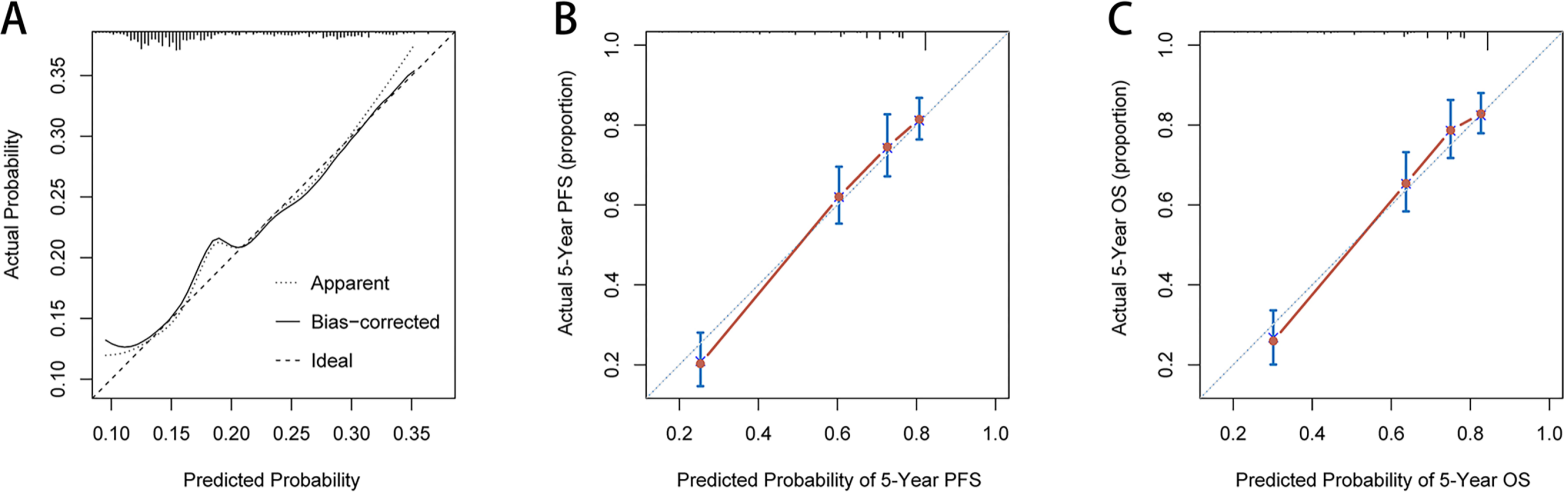
The calibration curves for predicting complication risk (A), progression-free survival (B) and overall survival (C) in CRC patients. The *X* axis presents the predicted probability and the *Y* axis shows the actual probability. The calibration lines fit along with the 45 reference Abbreviations: PFS, progression-free survival; OS, overall survival.

Based on the independent influence variables of complications identified in the multivariate logistic regression analysis, a nomogram was established to predict postoperative complication risks for CRC patients (Fig. 6A). Age was used as a continuous variable to improve the prediction accuracy of the nomogram. The C-index for the nomogram prediction was 0.625 (95% CI [0.568–0.682]). The calibration curves for the probability of postoperative complications showed good consistency between the prediction of the nomogram and the actual observations (Fig. 7A). Based on multivariate survival analyses, two nomograms were developed to predict 1–5 year PFS and 1–5 year OS for CRC patients (Figs. 6B, 6C). The C-index for PFS and OS prediction was 0.728 (95% CI [0.698–0.758]) and 0.729 (95% CI [0.696–0.762]), respectively. Calibration curves for the probability of 1–5 year PFS and 1–5 year OS showed optimal consistency between the prediction of ALI-based nomograms and actual observations (Figs. 7B, 7C).

## Discussion

Pro-inflammatory tumor microenvironments play important roles in cancer progression (Mantovani et al., 2008). There is increasing evidence to suggest that the elevation of some clinical inflammatory factors predicts prognoses in tumor patients, and that combinations of inflammatory factors further improves the efficacy of prognosis prediction. The NLR, lymphocyte to monocyte ratio (LMR), and Glasgow prognostic score (GPS) have been reported to be associated with clinical outcomes in patients with various cancers (Kubo et al., 2016; Lu et al., 2019; Tan et al., 2018). Recently, Jafri, Shi & Mills (2013). proposed a new ALI, which was based on BMI, albumin and NLR, to provide important prognostic information to cancer patients. Low ALI scores represent decreases in BMI, decreases in serum albumin and increases in serum NLR, indicating poor patient prognosis and high inflammatory responses.

Cancer patients are more likely to be malnourished because of the high metabolism and proliferation of tumor cells, leading to losses of muscle, fat and weight. Malnutrition is associated with impaired immune function; the body’s immune system fails to clear cancer cells, and increases the risk of tumor-related death (Tomita, Ayabe & Nakamura, 2017). BMI and serum albumin levels are the most commonly used indicators for assessing nutritional status, and are useful indicators for assessing the prognosis of colorectal cancer (Doleman et al., 2016; Gupta & Lis, 2010; Hu et al., 2019). In addition, other studies have found that albumin levels is associated with systemic inflammation during tumor proliferation and invasion; stimulated pro-inflammatory factors affect liver cell catabolism and anabolism, decreasing albumin levels in the body (Balkwill, 2009; Brenner, Blaser & Mak, 2015). An increase in NLR leads to neutrophilia or lymphopenia. Neutrophils produce cytokines that inhibit lymphocyte-mediated immune activity, consisting of natural killer T cells or activated T cells, while cytokine releasing lymphocytes play key roles in killing cancer cells, regulating cancer cell proliferation, apoptosis, angiogenesis and metastasis (Eerola, Soini & Pääkkö, 1999; Eerola, Soini & Pääkkö, 2000; Leffers et al., 2009; Ropponen et al., 1998; Zhang et al., 2019). Therefore, NLR may serve as host markers for tumor immune defenses, tumor cell proliferation and invasion of biological activity. ALI combines commonly used indicators of nutrition and inflammation in clinical practice, which not only reflects the nutritional status of tumor patients, but also reflects tumor-related inflammatory responses. ALI is therefore a comprehensive indicator for predicting the prognosis of various tumors. By comparing the AUC values, we found that ALI was better at predicting prognosis than its components (ALB, NLR and BMI) in both short-term and long-term outcomes. And by comparing the prognostic efficacy of K-M survival curve, we proved that ALI can be more effectively separated the survival curve.

The cut-off value of ALI is different for different tumors. The cut-off for advanced NSCLC is 18 (Jafri, Shi & Mills, 2013), for small-cell lung cancer it is 19.5 (He et al., 2015), for unresectable metastatic colorectal cancer it is 28.9 (Shibutani et al., 2019), and for NSCLC cancer the cut-off is 37.66 (Tomita, Ayabe & Nakamura, 2017), These values may be related to the extent of systemic inflammatory responses in different tumors. In our study, we are convinced that due to physical differences between men and women, it is more accurate to use gender-specific X-tile program to determine optimal prognostic cut-off values for ALI. Therefore, for CRC patients undergoing surgery, we determined the cut-off value of ALI for males is 31.6, and 24.4 for females.

From correlation analyses, men with advanced age, clinical distant metastasis, colon cancer, large tumor size and high CEA levels were more likely to have low ALI. Hence, ALI reductions may reflect more aggressive tumor characteristics and severe tumor-related inflammation. Malnutrition is associated with an increased incidence of postoperative complications (Hall, 2006). ALI, as a nutrition-related indicator, may be closely related to postoperative complications. In our study, approximately 19.2% of patients had different degrees of postoperative complications, and patients with low-ALI were more likely to have postoperative complications. Our multivariate logistic analyses showed that low ALI is an independent risk factor for postoperative complications in CRC patients. Research has shown that postoperative complications affects patient prognosis, and complication severity is related to the survival time of malignant tumors (Andalib et al., 2013). This may be stress related, in that stress responses caused by surgical complications significantly increase neutrophils, and elevated neutrophils are a high-risk factor for tumor growth and spread. Moreover, elevated circulating neutrophils, high tumor neutrophil infiltration, and high NLR are associated with a poor patient prognosis (Forget, Dinant & De Kock, 2015). To demonstrate factors that affect postoperative complications, we established a complication risk nomogram based on results from multivariate logistic analyses, to predict risks for postoperative complications in CRC patients. In this nomogram, the contribution of age and ALI increases with adverse stages, and patients with lower scores have lower risks of postoperative complications, than patients with higher scores. Data from the C-index and calibration plots confirmed that the nomogram had a medium prediction accuracy.

Through multivariate survival analyses, we found that ALI, pT stage, pN stage, clinical distant metastasis and CEA levels were independent predictors of CRC patient prognosis. In addition, in the subgroup survival analysis, we saw that ALI was an independent influencing factor in multiple subgroups, suggesting that ALI was good in predicting the prognosis of CRC patients. It is accepted that TNM staging is the most reliable criterion for assessing CRC patient prognosis. However, it has been reported that patients with the same TNM stage often have different prognoses, suggesting that other factors are required for more accurate prognoses (Xie et al., 2019). We conducted a stratified prognostic evaluation based on different TNM stages, and found that survival outcomes of different ALI groups were significantly different in patients with TNM III-IV stage, but there were no differences in patients with TNM I-II stage. Similarly, in the subgroup multivariate survival analysis, we found that ALI was more suitable for the prognosis assessment of CRC patients with TNM stage III-IV. This may be related to the following reasons. Advance tumor patients have higher tumor burden, and more likely to have tumor cell proliferation and invasion, and tumor neovascularization, which leads to Changes in the proportion of neutrophils and lymphocytes in the body. In addition, Advance tumor patients are prone to obstruction, bleeding, and decreased food intake, which reduces the nutritional status. This observation implies that ALI can further assist in assessing the prognosis of CRC patients at the same advance TNM stage. For these reasons, we are convinced that preoperative ALI is a reliable, objective, repeatable and inexpensive predictor for CRC patient prognosis, following surgical resection, and could in time be considered a routine clinical application. In addition, we found that patients with III-IV stage can benefit from postoperative chemotherapy, but there was no statistical significance, which might be related to the insufficient sample size in this study.

For convenience and intuitive use in clinical work, we inserted ALI, pT stage, pN stage, clinical distant metastasis, and CEA level into nomograms, based on the results of the multivariate survival analysis. From these nomograms, the contribution of these factors increased with adverse stages, and patients with lower scores had longer PFS or OS, than patients with higher scores. Results from the C-index and calibration plots confirmed that the ALI-based nomograms had good prediction accuracy. To some extent, these nomograms provide a scientific basis for preoperative nutrition interventions, postoperative treatments and follow-up strategies in CRC patients. Patients with higher scores may have tumors that are more aggressive, with higher tumor-related inflammatory reactions. These indicators may be used as references for further treatment before and after surgery, such as postoperative radiotherapy and chemotherapy. Similarly, these patients should undergo increased ALI monitoring after surgery.

Although a previous study has reported the prognostic value of ALI in CRC patients (Shibutani et al., 2019), this study only included patients with unresectable metastatic CRC and the follow-up time was short, which led to certain limitations. Our study included I-IV stage CRC patients following surgical resection and conducted a long-term follow-up with the median follow-up of 63 months, confirming that ALI is a useful indicator to predict the prognosis of CRC patients following surgical resection. Our study also has some limitations. Firstly, this is a retrospective, single-center study, therefore a large-scale population prospective approach is recommended for future studies. In addition, due to the lack of other inflammation-related indicators, such as C-reactive protein and Glasgow Prognostic Scores, the predictive efficacy of ALI could not be compared with other inflammation-related indicators. Finally, ALI-based nomograms were constructed based on a limited sample of patients, therefore these must be validated in larger, more widespread populations.

## Conclusion

Our study confirmed that ALI is an independent predictor of short and long-term outcomes in CRC patients following surgical resection, especially at advance tumor stages. The ALI-based nomograms can provide accurate and individualized prediction of complication risk and survival for CRC patients.

##  Supplemental Information

10.7717/peerj.10100/supp-1 Supplemental Information 1 Raw data

## Abbreviations

CRC: colorectal cancer
ALI: Advanced Lung Cancer Inflammation Index
ALB: albumin
NLR: neutrophils/lymphocytes ratio
BMI: Body Mass Index
PFS: progression-free survival
OS: overall survival

## Additional Information and Declarations

### Data Availability

The following information was supplied regarding data availability: The raw measurements are available as a Supplementary File.
